# Efficient error correction algorithms for gene tree reconciliation based on duplication, duplication and loss, and deep coalescence

**DOI:** 10.1186/1471-2105-13-S10-S11

**Published:** 2012-06-25

**Authors:** Ruchi Chaudhary, J Gordon Burleigh, Oliver Eulenstein

**Affiliations:** 1 Department of Computer Science, Iowa State University, Ames, IA 50011, USA; 2 Department of Biology, University of Florida, Gainesville, FL 32611, USA

## Abstract

**Background:**

Gene tree - species tree reconciliation problems infer the patterns and processes of gene evolution within a species tree. Gene tree parsimony approaches seek the evolutionary scenario that implies the fewest gene duplications, duplications and losses, or deep coalescence (incomplete lineage sorting) events needed to reconcile a gene tree and a species tree. While a gene tree parsimony approach can be informative about genome evolution and phylogenetics, error in gene trees can profoundly bias the results.

**Results:**

We introduce efficient algorithms that rapidly search local Subtree Prune and Regraft (SPR) or Tree Bisection and Reconnection (TBR) neighborhoods of a given gene tree to identify a topology that implies the fewest duplications, duplication and losses, or deep coalescence events. These algorithms improve on the current solutions by a factor of *n *for searching SPR neighborhoods and *n*^2 ^for searching TBR neighborhoods, where *n *is the number of taxa in the given gene tree. They provide a fast error correction protocol for ameliorating the effects of gene tree error by allowing small rearrangements in the topology to improve the reconciliation cost. We also demonstrate a simple protocol to use the gene rearrangement algorithm to improve gene tree parsimony phylogenetic analyses.

**Conclusions:**

The new gene tree rearrangement algorithms provide a fast method to address gene tree error. They do not make assumptions about the underlying processes of genome evolution, and they are amenable to analyses of large-scale genomic data sets. These algorithms are also easily incorporated into gene tree parsimony phylogenetic analyses, potentially producing more credible estimates of reconciliation cost.

## Introduction

The availability of large-scale genomic data from a wide variety of taxa has revealed much incongruence between gene trees and the phylogeny of the species in which the genes evolve. This incongruence may be caused by evolutionary processes such as gene duplication and loss, deep coalescence, or lateral gene transfer. The variation in gene tree topologies can be used to infer the processes of genome evolution. Gene tree - species tree (GT-ST) reconciliation methods seek to map the history of gene trees into the context of species evolution and thus potentially link processes of gene evolution to phenotypic changes and diversification. Yet these methods can be confounded by error in the gene trees, which also may cause incongruence between the gene and species topologies. We introduce efficient algorithms to correct gene tree topologies based on the gene duplication, duplication and loss, or deep coalescence cost models. The algorithms work by identifying the small rearrangements in the gene trees that reduce the reconciliation cost. They are extremely fast and thus amenable to analyses of enormous genomic data sets.

Perhaps the most commonly used and computationally feasible approach to GT-ST reconciliation is gene tree parsimony, which seeks to infer the fewest evolutionary events (e.g., duplication, loss, coalescence, or lateral gene transfer) needed to reconcile a gene tree and species tree topology [1]. This approach also can be extended to infer species phylogenies, finding the species tree that implies the fewest evolutionary events implied by the gene trees (e.g., [2-4]). However, the gene trees often are estimated using heuristic methods from short sequence alignments, and consequently, there is often much error in the estimated gene tree topologies. Error in the gene trees creates more GT-ST incongruence and can radically affect GT-ST reconciliation analyses, implying far more duplications, duplications and losses, or deep coalescence events than actually exist. For example, Rasmussen and Kellis [5] estimated that error in gene tree reconstruction can lead to 2-3 fold overestimates of gene duplications and losses. Gene tree error also can erroneously imply large numbers of duplications near the root of the species tree [6,7], and it can mislead gene tree parsimony phylogenetic analyses (e.g., [8-10]).

Several approaches have been proposed to address gene tree error in GT-ST reconciliation. First, questionable nodes in a gene tree or nodes with low support may be collapsed prior to gene tree reconciliation, and the resulting non-binary gene trees may be reconciled with species trees [11-13]. Similarly, GT-ST reconciliations can use a distribution of gene tree topologies, such as bootstrap gene trees, rather than a single gene tree estimate [6,14,15]. Both of these approaches may help account for stochastic error and uncertainty in gene tree topologies, but they do not explicitly confront gene tree error. Methods also exist to simultaneously infer the gene tree topology and the gene tree reconciliation with a known species tree [5,16]. While these sophisticated statistical approaches appear very promising, they are computationally intensive, and it is unclear if they will be tractable for large-scale analyses. Another, perhaps a more computationally feasible, approach is to allow a limited number of local rearrangements in the gene tree topology if they reduced the reconciliation cost [17,18].

Previously [17,18] described a method to allow NNI-branch swaps on selected branches of a gene tree to reduce the reconciliation cost. Following [17,18], we address gene tree error in the reconciliation process by assuming that the correct gene tree can be found in a particular neighborhood of the given gene tree. We describe this approach for the gene duplication, duplication and loss, and deep coalescence models, which identify the fewest respective events implied from a given gene tree and given species tree. This neighborhood consists of all trees that are within one edit operation of the gene tree. While [17,18] use Nearest Neighbor Interchange (NNI) edit operations to define the neighborhood, we use the standard tree edit operations SPR [19,20] and TBR [19], which significantly extend upon the search space of the NNI neighborhood. The SPR and TBR local search problems find a tree in the SPR and TBR neighborhood of a given gene tree, respectively, that has the smallest reconciliation cost when reconciled with a given species tree. Using the algorithm from Zhang [21] the best known (naïve) runtimes are *O*(*n*^3^) for the SPR local search problem and *O*(*n*^4^) for the TBR local search problem, where *n *is the number of taxa in the given gene tree. These runtimes typically are prohibitively long for the computation of larger GT-ST reconciliations. We improve on these solutions by a factor of *n *for the SPR local search problem and a factor of *n*^2 ^for the TBR local search problem. This makes the local search under the TBR edit operation as efficient as under the SPR edit operation, and it provides a high-speed gene tree error-correction protocol that is computationally feasible for large-scale genomic data sets.

We also evaluated the performance of our algorithms using the implementation of SPR based local search algorithms. Note, that the SPR neighborhood is properly contained in the TBR neighborhood for any given tree. Thus the performance of the SPR based program provides a conservative estimate of the performance of the TBR based program. We test our programs on a collection of 106 yeast gene trees, some of which contain hundreds of leaves, and we demonstrate how it can be easily incorporated into large-scale gene tree parsimony phylogenetic analyses.

## Basic notation and preliminaries

Throughout this paper, the term tree refers to a rooted full binary (phylogenetic) tree.

Let *T *be a tree. The leaf set of *T *is denoted by *Le*(*T*). The set of all vertices of *T *is denoted by *V*(*T*) and the set of all edges by *E*(*T*). The *root *of *T *is denoted by *Ro*(*T*). The set of internal vertices of *T *is *I*(*T*):= *V*(*T*)\*Le*(*T*).

Given a vertex *v *∈ *V*(*T*), we denote the *parent *of *v *by *Pa_T_*(*v*). Let *u *:= *Pa_T_*(*v*). The edge that connects *v *with *u *is written as (*u, v*). The first element in the pair is always the parent of the second element. The set of all children of *v *is denoted by *Ch_T_*(*v*) and the children are called *siblings*. For *w *∈ *Ch_T_*(*v*), the sibling of *w *is denoted by *Sb_T_*(*w*).

We define ≤*_T _*to be the *partial order *on *V*(*T*) where *x*≤*_T _y *if *y *is a vertex on the path between *Ro*(*T*) to *x*, and write *x *<*_T _y *if *x *≤ *_T _y *and *x *≠ *y*. The *least common ancestor *of a non-empty subset *L *⊆ *V*(*T*), denoted as *LCA_T_*(*L*), is the unique smallest upper bound of *L *under ≤*_T_*. Given *x, y *∈ *V*(*T*), *d_T_*(*x, y*) denotes the number of edges on the unique path between *x *and *y *in *T*.

Given *U *⊆ *V*(*T*), we denote by *T*(*U*) the unique rooted subtree of *T *that spans *U *with the minimum number of vertices. Furthermore, the *restriction *of *T *to *U*, denoted by *T*_|*U*_, is the rooted tree that is obtained from *T*(*U*) by suppressing all non-root vertices of degree two. The *subtree *of *T *rooted at *u *∈ *V*(*T*), denoted by *T_u_*, is defined to be *T*_|*U*_, for *U*:= {*v *∈ *Le*(*T*): *v *≤*_T _u*}. Two trees *T*_1 _and *T*_2 _are called *isomorphic *if there exists a bijection between the vertex sets of *T*_1 _and *T*_2 _which maps a vertex *u*_1 _of *T*_1 _to vertex *u*_2 _of *T*_2 _if the subtree rooted at *u*_1 _in *T*_1 _has the same leaf set as the subtree rooted at *u*_2 _in *T*_2_. If an isomorphism exists between *T*_1 _and *T*_2_, we write *T*_1 _≃ *T*_2_.

Given function *f *: *A *→ *B*, we denote by *f*(*A*') where *A*' ⊆ *A *a set of images of each element in *A*' under *f*.

### The reconciliation cost models

A *species tree *is a tree that depicts the branching pattern representing the divergence of a set of species, whereas a *gene tree *is a tree that depicts the evolutionary history among the sequences encoding one gene (or gene family) for a given set of species. We assume that each leaf of the gene tree is labeled with the species from which that gene was sampled. Let *G *be a gene tree and *S *a species tree. In order to compare *G *with *S*, we require a mapping from each gene *g *∈ *V*(*G*) to the most recent species in *S *that could have contained *g*.

**Definition 1 (Mapping**). *The *leaf-mapping ℒ*_G,S _*: *Le*(*G*) → *Le*(*S*) *is a surjection that maps each leaf g*∈ *Le*(*G*) *to that unique leaf s *∈ *Le*(*S*) *which has the same label as g. The extension *ℳ*_G,S _*: *V*(*G*) → *V*(*S*) *is the mapping defined by ℳ_G,S_*(*g*):= *LCA*(ℒ*_G,S_*(*Le*(*G_g_*))). *For convenience, we write *ℳ(*g*) *instead of *ℳ*_G,S_*(*g*) *when G and S are clear from the context*.

**Definition 2 (Comparability**). *Given trees G and S, we say that G is *comparable *to S if a leaf-mapping ℒ_G,S_(g) is well defined*.

Throughout this paper we use the following terminology: *G *is a gene tree that is comparable to the species tree *S *through a leaf-mapping ℒ*_G,S_*, and *n *is the number of taxa present in both input trees.

Now we define different reconciliation costs from *G *to *S *for a given mapping ℳ*_G,S _*that extends ℒ*_G,S_*. The reconciliation cost are based on the models of gene duplication [22,23], duplication-loss [21], and deep coalescence [21].

**Definition 3 (Duplication cost**).

• *The *duplication cost *from g *∈ *V*(*G*) *to S*, $C_{D}\left(G,S,g\right):=\left\{\begin{array}{c} 1,\;\;\;\;\;ifM\left(g\right)\in M\left(Ch\left(g\right)\right);\\ 0,\;\;\;\;otherwise. \end{array}\right)$

• *The *duplication cost *from G to S*, $C_{D}\left(G\text{,}\;S\right):=\sum_{g\in I\left(G\right)}C_{D}\left(G,S,g\right)$.

**Definition 4 (Duplication-loss cost**).

• *The *loss cost *from g *∈ *V*(*G*) *to S*,

$$C_{L}\left(G,S,g\right):=\left\{\begin{array}{cc} 0, & if\forall h\in Ch\left(g\right):M\left(g\right)=M\left(h\right);\\ \sum_{h\in Ch\left(g\right)}|d_{S}\left(M\left(g\right),M\left(h\right)\right)-1|, & otherwise. \end{array}\right)$$

• The duplication-loss cost *from G to S, *$C_{DL}\left(G,\;S\right):=\sum_{{{}_{g}}_{\in I\left(G\right)}}\left(C_{D}\left(G,S,g\right)\;+C_{L}\left(G,S,g\right)\right)$.

**Definition 5 (Deep coalescence cost**).

• *The *number of lineages from *g *∈ *V*(*G*) *to h *∈ *Ch*(*g*) *in S*,

$$C_{DC}\left(G,\;S,\;g\right):=\sum_{h\in Ch\left(g\right)}d_{S}\left(M\left(g\right),\;M\left(h\right)\right).$$

• *The *deep coalescence cost *from G to S*, $C_{DC}\left(G,S\right):=\sum_{{{}_{g}}_{\in I\left(G\right)}}C_{DC}\left(G,S,g\right)-|E\left(S\right)|$.

### The error-correction problems

Here we give definitions for tree rearrangement operations TBR [19] and SPR [19,20], and then formulate the Error-Correction problems that were motivated in the introduction.

**Definition 6 **(Tree Bisection and Reconnection (TBR)). *Let T be a tree. For this definition, we regard the *planted tree *Pl*(*T*) *as the tree obtained from adding the *root edge{*r, Ro*(*T*)} *to E*(*T*), *where r *∉ *V*(*T*).

Let *e *:= (*u, v*) ∈ *E*(*T*), *and X and Y be the connected components that are obtained by removing edge e from T such that v *∈ *X *and *u *∈ *Y. We define TBR_T _*(*v, x, y*) *for x ∈ X and y *∈ *Y to be the tree that is obtained from Pl*(*T*) *by first deleting edge e, and then adjoining a new edge f between X and Y as follows*:

1. *If x *≠ *Ro*(*X*) *then suppress Ro*(*X*) *and create a new root by subdividing edge *(*Pa*(*x*), *x*).

2. *Subdivide edges *(*Pa*(*y*), *y*) *by introducing a new vertex y'*.

3. *Re-connect components X and Y by adding edge f *= (*y', Ro*(*x*).

4. *Suppress the vertex u, and rename vertex y' as u*.

5. *Contract the root edge*.

**Figure 1:**
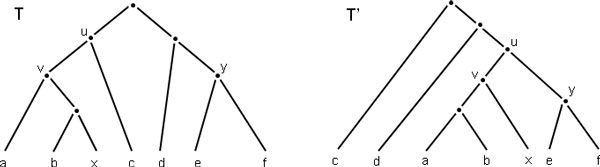
**An TBR operation**. Tree *T*' = TBR*_T_*(*v, x, y*) results from *T *after performing single TBR operation.

*We say that, the tree TBR_T _(v, x, y) is obtained from T by a *tree bisection and reconnection (TBR) *operation that bisects the tree T into the components X and Y , and reconnects them above the nodes x and y. (See *Figure 1*.) We define the following neighborhoods for the TBR operation*:

1. *TBR_G_*(*v, x*) *:= *∪ *_y∈Y _TBR_G_*(*v, x, y*)

2. *TBR_G_*(*v*) *:= *∪*_x∈X _TBR_G_*(*v, x*)

3. *TBR_G _:= *∪_(*u, v*)∈*E*(*G*) _*TBR_G_*(*v*)

**Figure 2:**
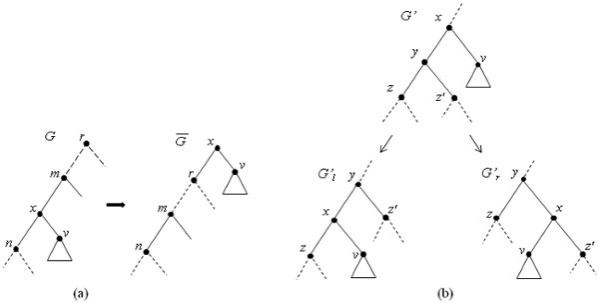
**The NNI adjacency graph**. (a) The tree $\bar{G}$ is obtained from *G *by pruning and regrafting subtree *G_v _*to the root of *G*. The vertex *x *∈ *V*(*G*) is suppressed, and the new vertex above root in $\bar{G}$ is named *x*. (b) Two NNI operations NNI*_G'_*(*z*') and NNI*_G'_*(*z*) produce left-child *G'_l _*and right-child *G'_r _*of *G*' in  X .

**Definition 7 **(Subtree Prune and Regrafting (SPR)). *The SPR operation is defined as a special case of the TBR operation. Let e *:= (*u, v*) ∈ *E*(*T*), *and X and Y be the connected components that are obtained by removing edge e from T such that v *∈ *X *and *u *∈ *Y. We define SPR_T _*(*v, y*) *for y *∈ *Y to be TBR_T _*(*v, v, y*). *We say that the tree SPR_T _*(*v, y*) *is obtained from T by performing subtree prune and regraft (SPR) operation that prunes subtree T_v _and regrafts it above y. (See *Figure 2(a).)

*We define the following *neighborhoods *for the SPR operation*:

*1. SPR_G_*(*v*) *:= *∪*_y∈Y _SPR_G_*(*v, y*)

*2. SPR_G _:= *∪_(*u, v*)∈*E*(*G*) _*SPR_G_*(*v*)

We now state the SPR based error-correction problems for duplication (D), duplication-loss (DL), and deep coalescence (DC). Let Γ ∈ {D, DL, DC}.

### Problem 1 (SPR based error-correction for Γ (SEC-Γ))

*Instance*: *A gene tree G and a species tree S*.

Find: A gene tree G* ∈ *SPR_G _such that *$C_{\Gamma }\left(G^{*},S\right)=\underset{G^{\prime }\in SPR_{G}}{\text{min}}C_{\Gamma }\left(G^{\prime },S\right)$.

The TBR based error-correction for Γ (TEC-Γ) problems are defined analogously to the SPR based error-correction for Γ (SEC-Γ) problems.

### Solving the SEC-Γ problems

In this section we study the SPR based error-correction problems, for duplication (D), duplication-loss (DL), and deep coalescence (DC), in more detail. Our efficient solution for these problems are based on solving restricted versions of these problems efficiently. For each Γ ∈ {*D, DL, DC*} we first define a restricted version of the SEC-Γ problem, which we call the restricted SPR based error-correction for the Γ (R-SEC-Γ) problem.

### Problem 2 (Restricted SPR based error-correction for (R-SEC-Γ))

*Instance: A gene tree G, a species tree S, and v *∈ *V*(*G*).

Find: A gene tree G* ∈ *SPR_G_*(*v*) *such that *$C_{\Gamma }\left(G^{*},S\right)=\underset{G^{\prime }\in SPR_{G}\left(v\right)}{\text{min}}C_{\Gamma }\left(G^{\prime },S\right)$.

**Observation 1**. Let Γ ∈ {*D, DL, DC*}. *Given a gene tree G and a species tree S*, the *SEC-*Γ *problem can be solved as follows: (i) solve the R-SEC-*Γ *problem for every v *∈ *V*(*G*) *where v *≠ *Ro*(*G*), (ii) under all solutions found return a minimum scoring gene tree G*.

Naïvely, the R-SEC-Γ problem can be solved in Θ(*n*^2^) time by computing the cost $C_{\Gamma }\left({\text{G}}^{\prime },\text{ S}\right)$ for each *G*' ∈ *SPR_G_*(*v*). The cost for a given gene and species tree can be computed in Θ(*n*) time [21]. We introduce a novel algorithm for the R-SEC-Γ problem that improves by a factor of *n *on the naïve solution. This speedup is achieved by semi-ordering the trees in *SPR_G_*(*v*), for each *v *∈ *V*(*G*), such that the score-difference of any two consecutive trees in this order can be computed in constant time.

### Ordering the trees in SPR*_G_*(*v*)

Consider a graph on trees in *SPR_G_*(*v*), in which every two adjacent trees are one NNI [19] operation apart. We show that such a graph is a rooted full binary tree, after providing necessary definitions.

**Definition 8 **(Nearest Neighbor Interchange (NNI)). *We define the NNI operation as a special case of the SPR operation. Let e *∈ *E*(*T*) *where e *:= (*u, v*), *and X and Y be the connected components that are obtained by removing edge e from T such that v *∈ *X and u *∈ *Y. We define NNI_T _*(*v*) *to be SPR_T _*(*v, y*) *for y:= Pa*(*u*), *and say that NNI_T _*(*v*) *is obtained from T by performing nearest neighbor interchange (NNI) operation that prunes subtree T_v _and regrafts it above the parent of v's parent. (See *Figure 2(b).)

**Definition 9 **(NNI distance). *Let the *NNI-distance, *denoted as d_NNI_*(*T*_1, _*T*_2_), *between two trees T*_1 _*and T*_2 _*over n taxa be the minimum number of NNI operations required to transform T*_1 _*into T*_2_.

**Definition 10 **(NNI-adjacency graph). *The *NNI-adjacency graph, *denoted as *$X=\left(V,E\right)$, *is the graph where V = SPR_G_*(*v*) *and *{*T*_1, _*T*_2_} ∈ *E if d_NNI_*(*T*_1, _*T*_2_) = 1.

**Lemma 1**.  X *is a tree*.

*Proof*. We prove it by showing that there exists a unique path between every two vertices in  X . Let *G', G*'' ∈ *V*( X ), thus *G', G*'' ∈ SPR*_G_*(*v*). Let *G*':= SPR*_G_*(*v, x*_1_) and *G*'':= SPR*_G_*(*v, x*_2_). We use induction on *d_G_*(*x*_1_, *x*_2_). Let *d_G_*(*x*_1_, *x*_2_) = 1 and assume without loss of generality that *x*_2 _= *Pa_G_*(*x*_1_). Thus, *G*'' = NNI*_G'' _*(*Sb*(*x*_1_)). So the hypothesis holds for *d_G_*(*x*_1_, *x*_2_) = 1. Assume now that the hypothesis is true for *d_G_*(*x*_1_, *x*_2_) ≤ *k *and suppose *d_G_*(*x*_1_, *x*_2_) = *k *+ 1. Since *G *is a tree, there must be a unique path between *x*_1 _and *x*_2_; let *y *be a vertex on this path. Let *d_G_*(*y, x*_1_) = 1, and *G^n ^*:= SPR*_G_*(*v, y*). If *y *= *Pa_G_*(*x*_1_), then *G^n ^*= NNI*_G'_*(*v*); otherwise *G^n ^*= NNI*_G'_*(*Sb*(*y*)). Since *d_G_*(*y, x*_2_) = *k*, thus (by induction hypothesis) the hypothesis is valid for *d_G_*(*x*_1_, *x*_2_) = *k *+ 1. □

**Theorem 1**.  X *is a rooted full binary tree*.

*Proof*. In view of Lemma 1, it suffices to show that except a unique vertex of degree 2 all other vertices in  X  are of degree 1 or 3. Let *G*' ∈ *V*( X ), thus *G*' = SPR*_G_*(*v, y*) for some *y *∈ *V*(*G*). There are three cases:

**Case 1: ***y ***is a root**. Let *y*_1 _∈ *Ch_G_*(*y*). Let *G*^1 ^:= SPR*_G_*(*v, y*_1_), thus $G^{\prime }=\text{NN}{\text{I}}_{{\text{G}}^{\text{1}}}\left(v\right)$). Hence {*G*^1^, *G*'} ∈ *E*( X ). Since |*Ch_G_*(*y*)| = 2, *G*' must be a degree 2 vertex in  X .

**Case 2: **y **is a leaf**. Let *y*_1 _= *Pa_G_*(*y*). Let *G*^1 ^:= SPR*_G_*(*v, y*_1_), thus *G*^1 ^= NNI*_G'_*(*v*). Hence {*G*^1^, *G*'} ∈ *E*( X ), and consequently, *G*' is a degree 1 vertex in  X .

**Case 3: ***y ***is an internal vertex**. Let *y*_1 _= *Pa_G_*(*y*) and *y*_2 _∈ *Ch_G_*(*y*). Let *G*^1 ^:= SPR*_G_*(*v, y*_1_), thus *G*^1 ^= NNI*_G'_*(*v*). Let *G*^2 ^:= SPR*_G_*(*v, y*_2_), thus *G*' = NNI*_G_^2^*(*v*). Since *y *has one parent and two children in *G*, thus *G*' is a degree 3 vertex in  X .

This completes the proof.

### The score difference of consecutive trees in  X 

To solve the R-SEC-Γ problems we traverse tree  X . Two adjacent trees in *V*( X ) are one NNI operation apart. We show that $C_{\Gamma }$ score of a tree can be computed in constant time from the LCA computation of its adjacent tree.

Let *e *:= (*G', G*'') be an edge in  X . Let *x *:= *Pa*(*v*), *y *:= *Sb*(*v*), and *z, z*' ∈ *Ch*(*y*) in *G*' (see Figure 2(b)). Without loss of generality, let *G*'':= NNI*_G'_*(*z*). (Observe, *G*'' is similar to $G_{r}^{{}^{\prime }}$ of Figure 2(b).)

**Lemma 2**. ℳ*_G'',S_*(*y*) = ℳ*_G',S_*(*x*).

*Proof*. From NNI operation, *v, z*' ∈ *Ch_G''_*(*x*) and *z, x *∈ *Ch_G''_*(*y*). Also, $G_{z}^{{}^{\prime }}≃G_{z}^{{}^{\prime \prime }},\;G_{z^{\prime }}^{{}^{\prime }}≃G_{z^{\prime }}^{{}^{\prime \prime }},G_{v}^{{}^{\prime }}≃G_{v}^{{}^{\prime \prime }}$, so $Le\left({\text{G}}_{y}^{{}^{\prime \prime }}\right)=Le\left({\text{G}}_{x}^{{}^{\prime }}\right)$. Thus, ${M_{G^{\prime }}}_{,S}\left(x\right)=\text{LCA}\left(L_{G^{\prime },S}\left(Le\left(G_{x}^{{}^{\prime }}\right)\right)\right)=\text{LCA}\left(L_{G^{\prime \prime },S}\left(Le\left(G_{y}^{{}^{\prime \prime }}\right)\right)\right)={M_{G^{\prime \prime }}}_{,S}\left(y\right)$. □

**Lemma 3**. ℳ*_G'',S_*(*w*) = ℳ*_G',S_*(*w*), *for all w *∈ *V*(*G*')\{*x, y*}.

*Proof*. For $g\in V\left(G_{v}^{{}^{\prime }}\right)\cup \left(G_{z}^{{}^{\prime }}\right)\cup \left(G_{z^{\prime }}^{{}^{\prime }}\right)$, since $G_{g}^{{}^{\prime }}≃G_{g}^{{}^{\prime \prime }}$, therefore ℳ*_G',S_*(*g*) = ℳ*_G'',S_*(*g*). Also, except for subtree $G_{x}^{{}^{\prime }}$, the rest of the tree remains the same in $G_{x}^{{}^{\prime \prime }}$. Thus by Lemma 2, ℳ*_G',S_*(*Pa_G' _*(*x*)) = ℳ*_G'',S_*(*Pa_G''_*(*y*)). Inductively, ℳ*_G',S_*(*g*) = ℳ*_G'',S_*(*g*), for all *g *∈ *V*(*G*')\*V*(*G'_x_*). □

**Lemma 4**. ℳ*_G''_,_S_*(*x*) = *LCA*(ℳ*_G',S_*(*v*), ℳ*_G', S_*(*z*')).

*Proof*. From Lemma 3, ℳ*_G'',S_*(*v*) = ℳ*_G',S_*(*v*) and ℳ*_G'',S_*(*z*') = ℳ*_G',S_*(*z*'). Thus, ℳ*_G'',S_*(*x*) = LCA(*ℳ_G'',S_*(*v*),ℳ*_G'',S_*(*z*')) = LCA(ℳ*_G',S_*(*v*),ℳ*_G',S_*(*z*')). □

**Lemma 5**. $C_{\Gamma }\left(G^{\prime \prime },S,g\right)=C_{\Gamma }\left(G^{\prime },S,g\right)$, *for all g *∈ *V*(*G*'')\{*x, y*} *and *Γ ∈ {*D, DL, DC*}.

*Proof*. The gene duplication and loss status of a vertex, and the number of lineages from a vertex to its children in *G*' can change in *G*'' if its mapping or mapping of any of its children changes in ℳ*_G'',S_*. From Lemma 3, and also, since ℳ*_G''_,_S_*(*w*) = ℳ*_G',S_*(*w*), for *w *∈ *Ch*(*Pa_G'_*(*x*)), must have $C_{\Gamma }\left(G^{\prime \prime },S,Pa_{G^{\prime }}\left(x\right)\right)=C_{\Gamma }\left(G^{\prime },S,Pa_{G^{\prime }}\left(x\right)\right)$. Thus the Lemma follows. □

Let *e *:= (*G', G*'') ∈ *E*( X ) and Γ ∈ {D, DL, DC}. We define $\Gamma e:=C_{\Gamma }\left(G^{\prime \prime },S\right)-C_{\Gamma }\left(G^{\prime },S\right)$ with respect to the given species tree *S*. Observe that this score can be negative too. We study how Γ*_e _*can be computed efficiently for each edge *e *in  X .

**Theorem 2**. $\Gamma_{e}=\underset{g\in \left\{x,y\right\}}{\sum }\left(C_{\Gamma }\left(G^{\prime \prime },\;S,\;g\right)-C_{\Gamma }\left(G^{\prime },\;S,\;g\right)\right)$.

*Proof *.$\begin{array}{c} \Gamma_{e}=C_{\Gamma }\left(G^{\prime \prime },S\right)-C_{\Gamma }\left(G^{\prime },S\right)=\underset{g\in V\left(G^{\prime \prime }\right)}{\sum }\left(C_{\Gamma }\left(G^{\prime \prime },S,g\right)-C_{\Gamma }\left(G^{\prime },S,g\right)\right)=\underset{g\in V\left(G^{\prime \prime }\right)\backslash \left\{x,y\right\}}{\sum }(C_{\Gamma }\left(G^{\prime \prime },S,g\right)-\\ C_{\Gamma }\left(G^{\prime },S,g\right))+\underset{g\in \left\{x,y\right\}}{\sum }\left(C_{\Gamma }\left(G^{\prime \prime },S,g\right)-C_{\Gamma }\left(G^{\prime },S,g\right)\right)=\underset{g\in \left\{x,y\right\}}{\sum }\left(C_{\Gamma }\left(G^{\prime \prime },S,g\right)-C_{\Gamma }\left(G^{\prime },S,g\right)\right). \end{array}$

□

**Definition 11**. *Let *$\bar{G}:=SPR_{G}\left(v,Ro\left(G\right)\right)$, *and let P_G' _be a path from *$\bar{G}$*to G*^' ^*in * X . *For G*^'^, *we define the *score-difference $\Gamma_{\bar{G},G\prime }$*as *$\Gamma_{\bar{G},G\prime }:=\underset{e\in E\left(P_{G\prime }\right)}{\sum }\Gamma_{e}$.

**Theorem 3**. *For given S, G, and v *∈ *V*(*G*), *the tree G*' ∈ *V*( X ) *is the output of a R-SEC*-Γ *problem iff *$\Gamma_{\bar{G},G^{\prime }}=min_{G”\in V\left(X\right)\;}\Gamma_{\bar{G},G”}$.

*Proof*. Let $\Gamma_{\bar{G},G^{\prime }}=min_{G^{\prime \prime }\in V\left(X\right)\;}\Gamma_{\bar{G},G^{\prime \prime }}$. We prove that *G*' is the output of R-SEC-Γ problem. Since ${\Gamma_{Ḡ,G}}_{{}^{\prime }}=\underset{e\in E\left({P_{G}}_{{}^{\prime }}\right)}{\sum }\Gamma_{e}=\Gamma \left(G^{\prime },\;S\right)-\Gamma \left(Ḡ,\;S\right)$, thus *G*' gives the minimum normalized $C_{\Gamma }$ score over all trees in *V*( X ). Hence, *G*' must be the output of the R-SEC-Γ problem. The other direction follows similarly. □

### The algorithm

We describe a general algorithm, called Algo-R-SEC-Γ, to solve the R-SEC-Γ problem for each Γ ∈ {*D, DL, DC*}. Initially Algo-R-SEC-Γ computes (i) the root vertex of the NNI-adjacency graph  X , which we call $\bar{G}$, by regrafting the subtree *G_v _*above the root of *G*, (ii) the LCA mapping from $\bar{G}$ to *S*, and (iii) the Γ score from $\bar{G}$ to *S*. Then recursively Algo-R-SEC-Γ computes the LCA mapping and Γ score for every vertex *G*' in  X  when the LCA mapping and Γ score of its parent vertex in  X  is known. Algorithm 1 details Algo-R-SEC-Γ.

### Algorithm 1 - Algo-R-SEC-Γ

***Input: **A gene tree G, a species tree S, and v *∈ *V*(*G*)

**Output: **A tree G* ∈ *SPR_G_*(*v*) *such that *$C_{\Gamma }\left(G^{*},\;S\right)={\text{min}}_{G^{\prime }\in SPR_{G}\left(v\right)}C_{\Gamma }\left(G^{\prime },\;S\right)$

*01. Compute *$\bar{G}$*by pruning G_v _and regrafting at Ro(G)*

*02. Compute LCA mapping *$M_{Ḡ,S}$

*03. Call *$C_{\text{G}}\left(\bar{G},S\right)\;=Algo-Comp-Score\left(\bar{G},S,M_{\bar{G},S}\right)$

*04. Set *$BestTree=\bar{G}$, *BestScore *= 0

*05. Set *$G^{\prime }=Ḡ$, $M_{G^{\prime },S}=M_{Ḡ,S}$, $C_{\Gamma }\left(G^{\prime },\;S\right)=C_{\Gamma }\left(Ḡ,\;S\right)$, ${\Gamma_{Ḡ,G}}_{\prime }=0$

*06. **For **each *$k\neq Ro\left({Ḡ}_{Sb\left(v\right)}\right)$*in preorder traversal of *${Ḡ}_{Sb\left(v\right)}$, ***do***

*07*.    ***If **not backtracking, **then***

*08.       Set x = Pa_G'_*(*v*), *y *= *Sb_G_*'(*v*)

*09.       Set G*'' = *NNI_G'_*(*Sb_G'_*(*k*))

*10.       Set *ℳ*_G''.S _*= ℳ*_G',S_*, ℳ*G'',_S_*(*y*) = ℳ*_G',S_*(*x*)

*11*.       ℳ*_G'',S_*(*x*) = *LCA*(ℳ*_G',S_*(*k*),ℳ*_G',S_*(*v*))

*12.       Call *$\Gamma_{\left\{G^{\prime },G”\right\}}=\sum_{h\in \left\{x,y\right\}}Algo-G-Score\left(G”,\;S,\;M_{G”,S},\;h\right)-Algo-G-Score\left(G^{\prime },\;S,\;M_{G^{\prime },S},\;h\right)$

*13*.       $\Gamma_{Ḡ,G”}={\Gamma_{Ḡ,}}_{G^{\prime }}+\Gamma_{\left\{G^{\prime },G”\right\}}$

*14*.       ***If***$\Gamma_{Ḡ,G”}<BestScore$, ***then***

*15.          Set BestTree *= *G*'', $BestScore=\Gamma_{Ḡ,G”}$

*16*.       ***Else***,

*17.          Set *$x\;=\;P{a_{G}}_{{}^{\prime }}\left(v\right),\;y\;=\;P{a_{G}}_{{}^{\prime }}\left(x\right)$

*18.          Set G*'' = *NNI_G'_*(*v*)

*19.          Set *$M_{G^{\prime \prime },S}=M_{G^{\prime },S},M_{G^{\prime \prime },S}\left(x\right)=M_{G\prime ,S}\left(y\right)$

*20.          Set *$M_{G^{\prime \prime },S}(y)=LCA\left(M_{G^{\prime },S}\left(Sb_{G^{\prime }}(x)\right),M_{G^{\prime },S}(k)\right)$

*21.          Call *$\Gamma_{\left\{G^{\prime \prime },G^{\prime }\right\}}=\sum_{h\in \left\{x,y\right\}}Algo-G\;-\;Score\left(G^{\prime },S,M_{G^{\prime },S},h\right)-Algo\;-\;G\;-\;Score\left(G^{\prime \prime },S,M_{G^{\prime \prime },S},h\right)$

*22.          Set *$\Gamma_{Ḡ,G^{\prime \prime }}=\Gamma_{Ḡ,G^{\prime }}\;-\Gamma_{\left(G^{\prime \prime },G^{\prime }\right)}$

*23.    Set *$G^{\prime }=G^{\prime \prime },M_{G^{\prime },S}=M_{G^{\prime \prime },S},\Gamma_{Ḡ,G^{\prime }}=\Gamma_{Ḡ,G^{\prime \prime }}$

24. **Return **BestTree

### Algorithm 2 - Algo-Comp-Score

***Input: **A gene tree G, a species tree S, and LCA mapping *ℳ*_G,S_*

***Output:***$C_{\Gamma }$(*G,S*)

01. score = 0

*02. **For **each g *∈ *I*(*G*) *in preorder traversal of G*, ***do***

*03*.    $Call\;score\;=\;score\;+\;Algo-G-Score\left(G,S,{M_{G}}_{,S},\;g\right)$

*04. **If ***Γ*is DC, **then***

*05.    score = score *- |*I*(*S*)|

06. **Return **score

### Algorithm 3 - Algo-G-Score

*Input: A gene tree G, a species tree S, LCA mapping *ℳ*_G_*_,S_*, and g *∈ *I*(*G*)

***Output:***$C_{\Gamma }$(*G, S, g*)

*01. **If ***Γ *is D, **then***

*02*.    ***If***ℳ(*g*) ∈ ℳ(*Ch*(*g*)), ***then***

*03*.       ***Return ****1*

*04. **ElseIf ***Γ*is DL, **then***

*05 *   $ls=\sum_{h\in Ch\left(g\right)}|dp\left(M\left(h\right)\right)-dp\left(M\left(g\right)\right)-1|$

*06*.    ***If***ℳ(*g*) ∈ ℳ(*Ch*(*g*)), ***then***

*07*.       ***Return ****ls + 1*

*08*. ***Else***

*09*.    ***Return ****ls*

*10. **Else **//*Γ *is DC*

*11 *   ***Return***$\sum_{h\in Ch\left(g\right)}|dp\left(M\left(h\right)\right)-dp\left(M\left(g\right)\right)|$

**Lemma 6**. *The R-SEC-*Γ *problem is correctly solved by Algo-R-SEC-*Γ.

*Proof*. Lemma 1-5 and Theorem 1-3 directly imply that in order to prove the correctness of algorithm Algo-R-SEC-Γ, it is sufficient to prove that it correctly returns *G*' of minimum $\Gamma_{Ḡ,G\prime }$ among all *G*' ∈ *V*( X ). We will show that algorithm Algo-R-SEC- accounts each *G*' ∈ *V*( X ), correctly computes $\Gamma_{Ḡ,G\prime }$ for Γ ∈ {*D, DL, DC*}, and returns the right *G*' as output.

From Definition 10, *V*( X ) = SPR*_G_*(*v*). In Algo-R-SEC-Γ, step 1 prunes subtree *G_v _*and regrafts it above the root of *G *to create $\bar{G}$. Step 5 sets *G*' to $\bar{G}$. The for-loop in step 6 traverses subtree ${Ḡ}_{Sb\left(v\right)}$ in preorder. For each traversed vertex $k\neq Ro\left({Ḡ}_{Sb\left(v\right)}\right)$, step 9 builds the tree *G*'':= SPR*_G_*(*v, k*) by applying NNI operation on the last build *G*''. Each for-loop iteration sets *G*' to the last build *G*'' in step 23. $\bar{G}$ and *G*''s constitute all the trees in *SPR_G_*(*v*).

For $\bar{G}$, step 2 computes the LCA mapping and step 5 sets $\Gamma_{Ḡ,G^{\prime }}$ to zero. Following Lemma 2-4 and Theorem 2, step 10 and 11 update the LCA of G'' and step 12 computes $\Gamma_{\left\{G^{\prime },G^{\prime \prime }\right\}}$ by calling algorithm Algo-G-Score. Depending on Γ ∈ {*D, DL, DC*}, there are three cases:

**Case 1: **Γ **is D**. Algo-G-Score returns 1, if the vertex g ∈ *V*(*G*'') maps to the same vertex in *S *as any of its children maps to, otherwise 0.

**Case 2: **Γ **is DL**. Algo-G-Score computes losses by applying the formula of Definition 4. Further, it adds 1 if there is a duplication.

**Case 3: **Γ **is DC**. Algo-G-Score, returns the number of lineages from g to each of its children *h *∈ *Ch*(*g*) in *S*. For each *h *∈ *Ch*(*g*), depth of ℳ(*g*) is subtracted from depth of ℳ(*h*) to count number of edges between ℳ(*g*) and ℳ(*h*).

In Algo-R-SEC-Γ, step 13 computes $\Gamma_{\bar{G},G^{\prime \prime }}$ by adding $\Gamma_{\bar{G,}G^{\prime }}$ and $\Gamma_{\left\{G^{\prime },G^{\prime \prime }\right\}}$. When backtracking, steps 17-22 are executed to restore the right *G*' to compute the next unique *G*'' ∈ *Ch*_ X _(*G*). This ensures that the correct $\Gamma_{Ḡ,G^{\prime }}$ is computed for each *G*' ∈ *V*( X ).

In Algo-R-SEC-Γ, step 4 sets $\bar{G}$ as the BestTree and $\Gamma_{Ḡ,\bar{G}}\;\text{ = }\;\text{0}$ as BestScore. Every time a new *G*'' ∈ *Ch*_ X _(*G*) is encountered, step 14 compares ${\Gamma_{Ḡ,}}_{G^{\prime \prime }}$ with BestScore, and updates BestTree with *G*'' of the minimum ${\Gamma_{Ḡ,}}_{G^{\prime \prime }}$. After the for-loop, step 24 returns the BestTree. □

**Lemma 7**. *The R-SEC*-Γ *and SEC-*Γ *problems can be solved in *Θ(*n*) *and *Θ(*n*^2^) *time, respectively*.

*Proof*. We will prove that the algorithm Algo-R-SEC-Γsolves the restricted SPR based error-correction problems for each Γ ∈ {*D, DL, DC*} in Θ(*n*) time. In Algo-R-SEC-Γ, step 1 takes constant time. Step 2 precomputes LCA values for species tree in O(n) time [24], and so, finds LCA mapping from $\bar{G}$ to *S *in *O*(*n*) time in bottom-up manner. Step 3 computes the duplication, duplication-loss or deep coalescence score of $\bar{G}$ and *S *by calling Algo-Comp-Score. In Algo-Comp-Score, step 1 and step 2 runs for *O*(1) and *O*(*n*) time, respectively. Step 3 calls Algo-G-Score in each iteration of for-loop. Algo-G-Score runs for *O*(1) time for Γ ∈ {*D, DL, DC*}.

When Γ is DC, steps 4 and 5 are further executed in Algo-Comp-Score for constant time. Thus in Algo-R-SEC-Γ, step 3 runs for *O*(*n*) time. Further, steps 4 and 5 take constant time. The loop of step 6 runs for Θ(*n*) time. If condition of step 7 is true, steps 8-10 executes in constant time. With precomputed LCA values from step 2, step 11 executes in constant time. Algo-G-Score runs for constant time for Γ ∈ {*D, DL, DC*}, and lets step 12 to execute in constant time. Further, steps 13-15 execute for constant time too. If the condition in step 7 is false, then steps 17-22 execute in constant time, similarly. Finally, step 23 runs for constant time, and hence, the R-SEC-Γ problem can be solved in Θ(*n*) time. From Observation 1, Algo-R-SEC-Γ is called Θ(*n*) time to solve SEC-Γ problem. Thus, the SEC-Γ problem can be solved in Θ(*n*^2^) time. □

### Solving the TEC-Γ problems

In this section we study the TBR based error-correction problems, for duplication (D), duplication-loss (DL), and deep coalescence (DC). More precisely, we extend our solution for the SEC-Γ problems to solve the TEC-Γ problems. A TBR operation can be viewed as an SPR operation, except that the pruned subtree can be rerooted before it is regrafted. Our speed-up for the SEC-Γ problems is achieved by observing that the scores Γof any re-rooted pruned subtree and its remaining pruned tree are independent of each other. We define the R-TEC-Γ problems for the TEC-Γ problems, as we defined the R-SEC-Γ problems for the SEC-Γ problems. We will show that the R-TEC-Γ problems can be solved by solving two smaller problems separately and combining their solutions.

**Definition 12**. *Let T be a tree and x *∈ *V*(*T*). *RR*(*T, x*) *is defined to be the tree T , if x *= *Ro*(*T*) *or x *∈ *Ch*(*Ro*(*T*)). *Otherwise, RR*(*T, x*) *is the tree obtained by suppressing Ro*(*T*), *and subdividing the edge *(*Pa*(*x*), *x*) *by the new root node*.

**Lemma 8**. *Given a tuple *〈*G, S, v*〉, and *G*'':= *TBR_G_*(*v, x, y*), *for x *∈ *V*(*G_v_*), *y *∈ *V*(*G*)\*V*(*G_v_*). *Then*, $C_{\Gamma }(G^{\prime }\prime ,\;S)\leq_{G^{\prime }\in TBR_{G}(v)}C_{\Gamma }(G^{\prime },\;S)\;iffC_{\Gamma }(RR(G_{v},\;x),\;S)\leq_{x^{\prime }\in V(G_{v}}{}_{)}C_{\Gamma }(RR(G_{v},\;x^{\prime }),\;S)$*and *$C_{\Gamma }({G^{\prime }}^{\prime },\;S)\leq_{G^{\prime }\in TBR_{G}(v}{{}_{,}}_{x)}C_{\Gamma }(G^{\prime },\;S)$.

Proof. (⇒) Let *G*^1 ^:= *TBR_G_*(*v, x*_1, _*y*), for *x*_1 _∈ *V*(*G_v_*), and *x*_1 _≠ *x*. Now observe that, $\forall g\in V\left(G\right)\backslash V\left(G_{v}\right),\;C_{\Gamma }\left(G^{\prime \prime },\;S,\;g\right)=C_{\Gamma }\left(G^{1},\;S,\;g\right)$ Also, let *G*^2 ^:= *TBR_G_*(*v, x, y*_1_), for *y*_1 _∈ *V*(*G*)\*V*(*G_v_*), and *y*_1 _≠ *y*. Observe that, $\forall g\in V\left(G_{v}\right),\;C_{\Gamma }\left(G^{\prime \prime },\;S,\;g\right)=C_{\Gamma }\left(G^{2},\;S,\;g\right)$. Thus, if *G*'' gives the minimum duplication, duplication-loss, or deep coalescence score among all trees in *TBR_G_*(*v*), then the score contribution of vertices in *V*(*G_v_*) and *V*(*G*)\*V*(*G_v_*) is independent. Now looking at vertices of *G*, the best score is achieved when *G_v _*is rooted at *x*, i.e. $C_{\Gamma }\left(RR\left(G_{v},\;x\right),\;S\right)\leq_{x^{\prime }\in V\left(G_{v}\right)}C_{\Gamma }\left(RR\left(G_{v},\;x^{\prime }\right),\;S\right)$; also the best score is achieved when *RR*(G_v, _*x*) is regrafted at *y*, i.e., $C_{\Gamma }\left(G^{\prime \prime },\;S\right)\leq_{G^{\prime }\in \text{TB}{\text{R}}_{G}\left(v,x\right)}C_{\Gamma }\left(G^{\prime },\;S\right).\;\left(⇐\right)$. This follows similarly. □

Lemma 8 implies that a tree in TBR*_G_*(*v*) with the minimum duplication, duplication-loss, or deep coalescence cost can be obtained by optimizing the rooting for the pruned subtree, and the regraft location, separately. A best rooting for the pruned subtree is linear time computable [17,25], and the solution to the R-SEC problem identifies a best regraft location in Θ(*n*) time. This allows to obtain a tree in TBR*_G_*(*v*) with the minimum duplication, duplication-loss, or deep coalescence cost by evaluating only Θ(*n*) trees. Thus the R-TEC-Γ problem can be solved in Θ(n) time. The TEC-Γ problem can be solved by calling the solution of R-TEC-Γ problem Θ(*n*) times, and Theorem 4 follows.

**Theorem 4**. *The TEC-*Γ *problem can be solved in *Θ(*n*^2^) *time*.

### Experimental results

We tested the performance of the gene tree rearrangement algorithms on a set of 106 gene alignments containing sequences from 8 yeast taxa from Rokas et al. [26]. There is a well accepted phylogeny for the yeast species, and the data set has been used to test algorithms for gene tree parsimony based on the deep coalescence problem [27,28]. We constructed maximum likelihood gene trees for each gene using RAxML-VI-HPC version 7.0.4 [29], the gene trees were rooted with the outgroup *Candida albicans*. We used the new error correction algorithms to examine how much a single SPR rearrangement in the gene tree reduces the reconciliation cost based on deep coalescence and also gene duplications and losses. Over all genes the SPR error correction reduced the total deep coalescence cost from 151 to 53 (Table 1) and the duplication and loss cost from 481 to 175 (Table 2). Both the algorithms took only seconds to run for all 106 genes on a standard laptop.

**Table 1:** Error correction based on deep coalescence model

Reconciliation Cost	Original	Post-Correction
0	45	77
1	32	15
2	6	8

3	9	5
4	8	0
>4	6	1

The number of yeast gene trees with different reconciliation costs based on the deep coalescence model both before (Original) and after (Post-Correction) the SPR error correction.

**Table 2:** Error correction based on duplication and loss model

Reconciliation Cost	Original	Post-Correction
0	45	77
1-5	32	15
6-10	15	13

11-15	8	0
16-20	5	1
>20	1	0

The number of yeast gene trees with different reconciliation costs based on the duplication and loss model both before (Original) and after (Post-Correction) the SPR error correction.

We also implemented a protocol to use the gene rearrangement algorithm to correct for gene tree error in gene tree parsimony phylogenetic analyses. We first took a collection of input gene trees and performed a SPR species tree search using Duptree [30], which seeks the species tree with the minimum gene duplication cost. We used the duplication only cost (instead of duplications and losses) because when there is no complete sampling of all existing genes, the loss estimates may be inflated by missing sequences. After finding the locally optimal species tree, we used our SPR gene tree rearrangement algorithm to find gene tree topologies with a lower duplication cost. We then performed another SPR species tree search using Duptree, starting from the locally optimal species tree and using the new gene tree topologies. This search strategy is similar to re-rooting protocol in Duptree, which checks for better gene tree roots after a SPR species tree search [30,31]. We tested this protocol on data set of 6,084 genes (with a combined 81,525 leaves) from 14 seed plant taxa. This is the same data set used by [31], except that all gene tree clades containing sequences from a single species were collapsed to a single leaf. Our original SPR tree search found a species tree with 23,500 duplications. The SPR tree search after the gene tree rearrangements identified the same species tree, but the new gene trees had a reconciliation cost of only 18,213. This tree search protocol took just under 4 hours on a Mac Powerbook with a 2 GHz Intel Core 2 Duo processor and 2 GB memory.

## Conclusion

GT-ST reconciliation provides a powerful approach to study the patterns and processes of gene and genome evolution. Yet it can be thwarted by the error that is an inherent part of gene tree inference. Any reliable method for GT-ST reconciliation must account for gene tree error; however, any useful method also must be computationally tractable for large-scale genomic data. We introduce fast and effective algorithms to correct error in the gene trees. These algorithms, based on SPR and TBR rearrangements, greatly extend upon the range of possible errors in the gene tree from existing algorithms [17,18], while remaining fast enough to use on data sets with thousands of genes. These algorithms can correct trees based on a broad variety of evolutionary factors that can cause conflict between gene trees and species trees, including gene duplication, duplications and losses, and deep coalescence.

Our analysis on 106 yeast gene trees demonstrates that even a single SPR correction on the gene trees can radically improve upon the reconciliation cost. Our results in the yeast analysis are very similar to the 2-3 fold improvement in implied duplications and losses reported from the parametric gene tree estimation and reconciliation method of Rasmussen and Kellis [5]. However, in contrast, to this computationally complex method, the gene tree rearrangement algorithm is extremely fast, does not require assumption about the rates of duplication and loss, and is also amenable to corrections based on deep coalescence and duplications as well as duplications and losses. We do not claim that the gene correction algorithms produce a more accurate reconciliation than these parametric methods. However, they do present an extremely fast and flexible alternative.

We also demonstrated that this error correction protocol could easily be incorporated into a gene tree parsimony phylogenetic analysis. Previous studies have emphasized that gene tree parsimony is sensitive to the topology of the input trees. For example, the species tree may differ whether the gene trees are made using parsimony or maximum likelihood [8,10]. In our study, although the gene tree rearrangement did not affect the species tree inference, it did greatly reduce the gene duplication reconciliation cost.

While the results of the experiments are promising, they also suggest several directions for future research. First, further investigation is needed to characterize the effects of error on gene tree topologies. For example, it seems likely that gene tree errors may extend beyond a single SPR or TBR neighborhood. Yet, if we allow unlimited rearrangements, the gene trees will simply converge on the species tree topology. One simple improvement may be to weight the possible gene tree rearrangements based on support for different clades in the gene tree. Thus, well-supported clades may be rarely or never be subject to rearrangement, while poorly supported clades may be subject to extensive rearrangements. Finally, these approaches implicitly assume that all differences between gene trees and species trees are due to either coalescence, duplications, or duplications and losses. Future work will seek to combine these objectives and also address lateral transfer.

## Competing interests

The authors declare that they have no competing interests.

## Author's contributions

RC was responsible for algorithm design and program implementation, and wrote major parts of the paper. JGB performed the experimental evaluation and the analysis of the results, and contributed to the writing of the paper. OE supervised the project, contributed to the algorithmic design and writing of the paper.

All authors read and approved the final manuscript.
